# Evaluation of Antimycobacterial and Synergistic Activity of Plants Selected Based on Cheminformatic Parameters

**DOI:** 10.29252/.22.6.401

**Published:** 2018-11

**Authors:** Nafise Rahgozar, Gholamreza Bakhshi Khaniki, Soroush Sardari

**Affiliations:** 1 Department of Agricultural Biotechnology Engineering, Payam-e-Noor University, Tehran 19395, Iran; 2 Drug Design and Bioinformatics Unit, Medical Biotechnology Department, Biotechnology Research Center, Pasteur Institute of Iran, Tehran 13164, Iran

**Keywords:** Medicinal plants, *Mycobacterium*, Tuberculosis

## Abstract

**Background:**

Drug resistance is a major public health problem and a threat to progress made in bovine tuberculosis care and control worldwide. This study aimed at evaluating anti-mycobacterial and synergistic activity of some medicinal plants that were selected by cheminformatics studies against *Mycobacterium bovis*.

**Methods:**

Considering the strong synergistic antimycobacterial action of oleanolic acid in combination with tuberculosis drugs, NCBI database was explored to find the compounds with over 80% similarity to oleanolic acid, called S1. Plants containing S1-type compounds were traced to and resulted in five plants, including *Datura stramonium*, *Boswellia serrata Lavandula stoechas, Rosmarinus officinalis*, and *Thymus vulgaris*, as experimental samples. Crude extracts were prepared by percolation using 80% ethanol or as the product of a pharmaceutical company. The extracts were screened against *Mycobacterium bovis* using broth microdilution method and Alamar Blue Assay. Extracts from these plants were used in combination with isoniazid and ethambutol to investigate the possibility of synergy with respect to antimycobacterial activity.

**Results:**

The extracts from *D. stramonium*, *B. serrata* a, *L. stoechas*, *R. officinalis*, and *T. Thymus vulgaris* showed antimycobacterial activity of 375, 125, 250, 187.5, 500 µg/ml, respectively. The best synergistic results were for *L. stoechas* and *D. stramonium* in combination with ethambutol, the fractional inhibitory concentration index was 0.125 µg/ml for both.

**Conclusion:**

The observed antimycobacterial and synergistic activities are completely novel and obtained from targeted screening designed according to cheminformatics strategy. As for the synergistic action of the extracts, they could be used as supplements in bTB treatment.

## INTRODUCTION

In recent years, the number of people infected with bovine tuberculosis (bTB) has increased[1]. *Mycobacterium bovis* is the agent of bTB in a wide range of animal species and humansand results in a loss of three billion dollars annually worldwide. bTB is a disease that can infect cattle and human[2] and is an old disease since the ancient times. Today, bTB is considered as an infectious disease in developing countries with a great cause of mortality among people[2]. Currently, one third of the world population is infected with various forms of TB[3], and each year 2-3 million people of the world die from TB infection or its complications[4]. Based on an estimate, nearly one billion people are affected by the disease between 2000 and 2020[5].

HIV increases the risk of developing active TB, and this makes the treatment of TB difficult for the patients[6]. Unexpected drug resistance is one of the obstacles to the treatment of different kinds of TB. In some cases, the patients show resistance only to one of the antimycobacterial drugs. In some other cases, the contracted human or animal indicates resistance to two or more antimycobacterial drugs that can be the sign of existing multidrug-resistant species such as multidrug-resistant bTB and extensively drug-resistant bTB[7]. Unfortunately, the available antimycobacterial drugs are clearly limited and sometimes inefficient. Therefore, global efforts are required to control bTB among all sectors of society. According to recent surveys, plant products, as antimycobacterial agents, are the source of diverse and useful extracts and compounds[8]. Natural products are important sources of new antibiotics, and that is because of their amazing chemical variety. Also, natural products have been used and validated in traditional medicine during many centuries[9]. Typically, the secondary metabolites of plants can be employed to fight for various environmentally originated infection[10]. Due to the large population of plants, targeted screening is important to save time and research cost. In addition to traditional medicine, cheminformatics can be used to find the possible extracts easier and faster. One of the methods to explore chemical space in natural products for the purpose of drug discovery is gathering the information about biologically active chemical compounds and correlated their structure and activities in relational datasets[11].

A new approach in the current paper is to apply cheminformatics rational in order to select and consider traditionally used plants as a synergistic complementation for the conventional anti-mycobacterial drugs. The following plants are considered in this paper. *Datura stramonium* is a medicinal plant that has been used as analgesic and has been shown to have numerous alkaloids[12]. Lavender belongs to the family Lamiaceae and is a herbaceous, an aromatic and an evergreen plant[13]. Aerial parts of *Lavandula stoechas* have stronger antimicrobial effect than other parts of the plant[14]. It has been found that the leaves of this plant contain diterpene, large amounts of cyclic alcohols, flavonoids, and saponins. Among these, saponins have effective antibacterial properties[15]. *Boswellia serrata* is an aromatic resin plant that is obtained from several species of the genus *Boswellia*[16], a family of Burseraceae[17]. *B. serrata* generally contains 25-35% gum that is insoluble in alcohol and 60-70% of resin, and the rest includes some kinds of essential oils[18]. Boswellic acid is a series of pentacyclic triterpene and the main material in the resin of *Boswellia*[19]. *Rosmarinus officinalis* belongs to Lamiaceae family with green, sharp and fragrant leaves. Rosemary essential oil has antimicrobial and antioxidant properties that has been proven in several studies[20]. *Thymus vulgaris* is a member of Lamiaceae family that grows like a shrub with thin, reciprocal leaves and white flowers[21]. Thyme essential oil has antibacterial, antioxidant and antifungal properties[22].

In this study, we aim to investigate the secondary metabolite chemical space of plants by using the cheminformatic methods and trace compounds existing in medicinal plants that have synergistic effects on antimycobacterial drugs and also to examine the extract of such plants for the mentioned activity.

## MATERIALS AND METHODS

### Bioinformatics studies

By performing a comprehensive literature search on natural compounds with synergistic properties on bTB drugs, oleanolic acid was found as an antimycobacterial plant compound with the synergistic effect, when combining with antimycobacterial drugs[23]. In National Center of Biotechnology Information (NCBI) database (https://pubchem.ncbi.nlm.nih.gov/), we searched compounds with more than 80% similarity to oleanolic acid. Then the plants containing similar compounds with oleanolic acid were searched. Finally, we found five plants as candidates for experimental examinations.

### Preparation of powder and extraction of plants

Lavender, thyme, and rosemary plant extracts were prepared from Ebn-e-Masouyeh Pharmaceutical Company in Tehran, Iran. *Boswellia* and *Datura* plants, prepared from a herbal market in Tehran, were powdered with an electric mill. Percolation method was used to extract the plants. At first, 100 g of the powdered herb was transferred to the separatory funnel, and then 300 ml of 80% ethanol was added. The mixture was remained at room temperature (25 °C) for 24 hours, and gradual percolation was performed. This process was repeated three times at room temperature (25 °C) in different days. The extracts were concentrated by using RV05 rotary evaporator from IKA Co. (Germany) at ambient temperature[24].

### Minimum inhibitory concentration (MIC) determination of plant extracts and drugs

MIC test was conducted for the plant extracts and for antimycobacterial drugs by using microdilution assay[25]. Culture medium was the Middlebrook 7H9 broth from HiMedia, India. It was prepared according to the instructions on the container. In detail, 450 ml of distilled water was added to 3.5 g of the powder, and 2 mL of glycerol was added[26]. Culture medium was sterilized by autoclave and then was refrigerated immediately after preparation. The culture medium requires addition of a supplement called Middlebrook OADC (oleaic acid, albumin, dextrose, and catalase), which was prepared separately, and at the time of testing was added to the culture medium. OADC was prepared using 0.85 g of sodium chloride, 0.06 ml of oleic acid, 2 g of dextrose, 5 g albumin, 3 mg catalase, and 100 ml of distilled water.

The test compounds were prepared at the appropriate concentrations. Then, 1 mg of the drug was dissolved in 1000 µl of solvent (usually dimethyl sulfoxide), or 10 mg of extract was dissolved in 1000 µl of the solvent. At the beginning of the MIC test, 100 µl of culture medium was added to each well of a 96-well plate. An amount of 80 µl of culture medium was added to the first row of the plate, and then 20 µl of each compound (extracts, ethambutol as a positive control, dimethyl sulfoxide as a negative control) were added to the first cell in each column. After performing the serial dilution, 100 µl of BCG suspension (from BCG vaccine) equivalent to half McFarland were added to each cell, except the negative control column. The extract concentration tested was in the range of 500 to 1.95 µg/ml. The plates were incubated at 37 °C for four days[27]. After this time, Alamar blue solution was added to the culture medium. The plate was incubated at 37 °C, and MIC test results were read after 48 and 72 hours[28]. To read the MIC test result, the last cell that showed no color change was considered as MIC[29].

### Investigation of the synergistic effect using MIC test

To perform the test, half of the MIC of drugs was added to the culture medium. The rest of the test was done based on the previously explained steps. The fractional inhibitory concentration (FIC) was obtained using the following formulation and interpreted as synergy for FIC ≤ 0.5, no interaction for FIC = 1, and antagonism for FIC ≥ 2[23].


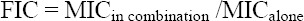


## RESULTS AND DISCUSSION

By using cheminformatics knowledge, similar antimycobacterial compounds to the template active compound were found. The results of the search for similar compounds of oleanolic acid are listed in Table 1. The results of plants containing similar compounds of oleanolic acid are illustrated in Table 2. The MIC tests were conducted to determine the MIC of plant extract and drugs that *Mycobacterium* could not grow at that concentration. The MIC test result for isoniazid and ethambutol drugs were 0.62 and 0.31 µg/ml, respectively. The synergistic effects of plant were determined by combining plant extracts with typical antimycobacterial drugs, isoniazid, and ethambutol. The results are indicated in Table 3. As shown in the Table, MIC test results showed that *B. serrata*, *D. stramonium*, *L. stoechas*, *R. officinalis*, and *T. vulgaris* had antimycobacterial activity. Among these, *B. serrata* and *T. vulgaris* had the highest and the lowest antimycobacterial activity, respectively. The result of combining plant extracts with ethambutol demonstrated the MIC of 125 µg/ml for *B. serrata* alone and 62.5 µg/ml in combination with ethambutol, as well as the FIC value of 0.5 µg/ml, which indicates the synergy activity. The MIC values of *L. stoechas* alone and in combination with ethambutol were 250 µg/ml and 31.3 µg/ml, respectively with a FIC value of 0.125, suggesting a strong synergistic activity. The MICs of *D. stramonium, T. vulgaris*, and *R. officinalis* alone were 375, 500, and 187.5 µg/ml and in combination with ethambutol were 46.88, 250, and 31.3 µg/ml, and their FICs were 0.125, 0.5, and 0.166 µg/ml, respectively, which shows the synergistic activities.

**Table 1:** The results of search for similar compounds with oleanolic acid

Compound name	Molecular formula	Molecular weight (g/mole)	Molecular structure
Glycyrrhetinic acid	C_30_H_48_O_3_	470.68384	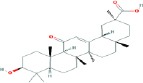
Ursolic acid	C_30_H_48_O_3_	456.70032	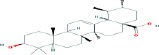
Boswellic acid	C_30_H_48_O_3_	456.70032	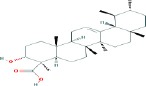
Maslinic acid	C_30_H_48_O_4_	472.69972	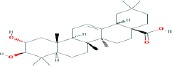
Asiatic acid	C_30_H_48_O_5_	488.69912	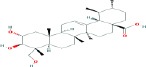
Hederagenin	C_30_H_48_O_4_	472.69972	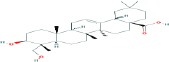
Echinocystic acid	C_30_H_48_O_4_	472.69972	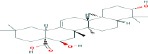
Maprounic acid	C_30_H_48_O_3_	456.70032	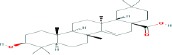
Pomolic acid	C_30_H_48_O_4_	472.69972	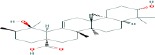
Rotundic acid	C_30_H_48_O_5_	488.69912	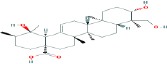
Sumaresinolic acid	C_30_H_48_O_4_	472.69972	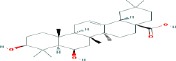
Augustic acid	C_30_H_48_O_4_	472.69972	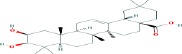
Medicagenic acid	C_30_H_46_O_6_	502.68264	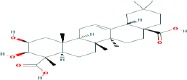
Euscaphic acid	C_30_H_48_O_5_	488.69912	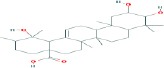
Quillaic acid	C_30_H_46_O_5_	486.68324	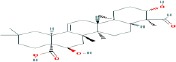
Daturaolone	C_30_H_48_O_2_	440.70092	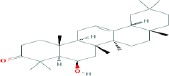
Sericic acid	C_30_H_48_O_6_	504.69852	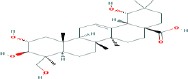
Wilforlide A	C_30_H_46_O_3_	454.68444	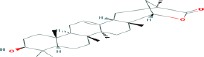
Bassic acid	C_30_H_46_O_5_	486.68324	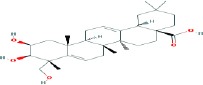
Barbinervic acid	C_30_H_48_O_5_	488.69912	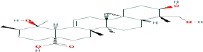

**Table 2:** Plants containing the similar compounds to oleanolic acid

Chemical compound	Plant with similar compound
Glycyrrhetinic acid	*Glycyrrhiza glabra*
Ursolic acid	*Ocimum basilicum* L.
	*Mentha piperita*
	*Origanum majorana*
	*Prunus subg. Prunus*
	*Lavandula*
	*Rosmarinus officinalis*
	*Thymus vulgaris*
Boswellic acid	*Boswellia*
Maslinic acid	*Olea europaea*
Asiatic acid	*Centella asiatica*
Hederagenin	*Sapindus mukorossi*
Echinocystic acid	*Codonopsis lanceolata*
	*Gleditsia sinensis* Lam.
	*Eclipta prostrata*
Maprounic acid	*Maprounea africana*
Pomolic acid	*Cecropia pachystachya*
Rotundic acid	*Ilex rotunda*
Sumaresinolic acid	*Warszewiczia coccinea*
Augustic acid	*Ambroma augusta*
Medicagenic acid	*Medicago sativa*
Euscaphic acid	*Rosa rugosa*
Quillaic acid	*Saponaria officinalis*
Daturaolone	*Datura stramonium*
Sericic acid	*Vochysia divergens*
Wilforlide A	*Tripterygium regelii*
Bassic acid	*Eucalyptus camaldulensis*
Barbinervic acid	*Clethra barbinervis*

**Table 3:** The result of MIC test for plants and the determined synergistic effect

Plant/drug	Individual MIC of plant extract (µg/ml)	Combination MIC (µg/ml)	Combined FIC index (µg/ml)	Synergy
*B. serrata*/EMB	125.0	62.50	0.500	Sy
*L. stoechas*/EMB	250.0	31.30	0.125	Sy
*D. stramonium*/EMB	375.0	46.88	0.125	Sy
*T. vulgaris*/EMB	500.0	250.00	0.500	Sy
*R. officinalis*/EMB	187.5	31.30	0.166	Sy
*B. serrata*/INH	125.0	31.30	0.250	Sy
*L. stoechas*/INH	250.0	62.50	0.250	Sy
*D. stramonium*/INH	375.0	250.00	0.660	N
*T. vulgaris*/INH	500.0	125.00	0.250	Sy
*R. officinalis*/INH	187.5	125.00	0.660	N

EMB, ethambutol; INH, isoniazid; MIC, minimum inhibitory concentration; FIC, fractional inhibitory concentration; Sy, synergistic; N, no interaction

The MIC test result of combining plant extracts with isoniazid showed the MIC value of 125, 250, 500 µg/ml for *B. serrata*, *L. stoechas*, and *T. vulgaris*when used alone and of 31.3, 62.5, 125 µg/ml in combination, respectively. They all had a similar FIC equal to 0.25 µg/ml and showed a synergistic activity. However, the FICs of *D. stramonium* and *R. officinalis* were equal to 0.66 µg/ml, which displays no interacting activity. Therefore, regarding the antimycobacterial and synergistic action of the mentioned plants, we can draw the conclusion that they could be used for supplementation along with regular drugs in bTB treatment. The results of a study by a group of researchers in 2012 showed that *Knowltonia vesicatoria*, as a traditional plant in South Africa, has antimycobacterial activity. They also observed a synergistic activity when the plant extract is combined with isoniazid drug[30]. These results are consistent with ours that indicated the synergistic effect of the *Boswellia* and *Lavandula* in combination with isoniazid. A previous study has also exhibited that 8-methoxypsoralen, a natural compound in many plant species, has antimycobacterial activity. This compound if combined with isoniazid, rifampin, and ethambutol drugs shows a synergistic effect against *Mycobacterium*[31]. In our study, the plant extracts of *Datura*, *Boswellia*, and *Lavandula* genera showed synergistic effects in combination with ethambutol and isoniazid. Another study by Bapelaa *et al*[32] has suggested that 7-methyljuglone and naphthoquinone isolated from *Euclea natalensis* roots show a synergistic effect when combining with isoniazid and rifampicin. They reported FIC indexes of 0.2 and 0.5 µg/ml, respectively. We, however, obtained FIC indexes of 0.5, 0.125, and 0.125, respectively for *Boswellia*, *Lavandula*, and *Datura* in combination with ethambutol and 0.25 µg/ml for both *Boswellia* and *Lavandula* in combination with isoniazid. The novel antimycobacterial results and the advanced synergistic activity observed for the mentioned plants shows the efficacy of our applied new cheminformatics-based strategy in designing a targeted screening and enhancing the chance of drug discovery programs in finding hit and lead compounds.

## References

[1] Garnier T, Eiglmeier K, Camus JC, Medina N, Mansoor H, Pryor M, Duthoy S, Grondin S, Lacroix C, Monsempe C, Simon S, Harris B, Atkin R, Doggett J, Mayes R, Keating L, Wheeler PR, Parkhill J, Barrell BG, Cole ST, Gordon SV, Hewinson RG (2003). The complete genome sequence of*Mycobacterium bovis*. Proceedings of the national academy of sciences USA.

[2] Bryde L, Waheed U (2013). Infectious diseases in developing countries. Scholars journal of applied medical sciences.

[3] Ghaffari-Fam S, Hosseini SR, Heydari H, Vaseghi-Amiri R, Daemi A, Sarbazi E, Nikbakht HA (2015). Epidemiological patterns of Tuberculosis disease in the Babol, Iran. Journal of analytical research in clinical medicine.

[4] Newton SM, Lau C, Gurcha SS, Besra GS, Wright CW (2002). The evaluation of forty-three plant species for *in vitro* antimycobacterial activities;isolation of active constituents from *Psoralea corylifolia* and *Sanguinaria canadensis*. Journal of ethnopharmacology.

[5] Zignol M, Gemert WV, Falzon D, Jaramillo E, Raviglione LBM (2011). Modernizing surveillance of antituberculosis drug resistance:from special surveys to routine testing. Clinical infectious diseases.

[6] Center for Disease Control and Prevention (CDC). HIV and tuberculosis (2013). Center for Disease Control and Prevention.

[7] KNCV [Internet] Tuberculosis MDR-TB and XDR-TB. October 2011.

[8] Arya V (2011). A review on anti-tubercular plants. International journal of pharmtech research.

[9] Dewick PM (2009). Medicinal Natural Products, A Biosynthetic Approach.

[10] Guzman JD, Gupta A, Evangelopoulos D, Basavannacharya C, Pabon LC, Plazas EA, Muñoz DR, Delgado WA, Cuca LE, Ribon W, Gibbons S, Bhakta S (2010). Anti-tubercular screening of natural products from Colombian plants:3-methoxynordomesticine, an inhibitor of MurE ligase of *Mycobacterium tuberculosis*. Journal of antimicrobial chemotherapy.

[11] Wikipedia (2016). Bioinformatics.

[12] Schulman ML, Bolton LA (1998). Datura seed intoxification in two horses. Journal of the South African veterinary association.

[13] Umezu T, Nagano K, Kosakai K, Sakaniwa M, Morita M (2006). Anticonflict effect of lavender oil and identification of its active constituents. Pharmacology biochemistry and behavior.

[14] Ozcan M (2003). Antioxidant activities of rosemary, sage, and sumac extracts and their combinations on stability of natural peanut oil. Journal of medicinal food.

[15] Kim HM, Cho SH (1999). Lavender oil inhibits immediatetype allergic reaction in mice and rats. Journal of pharmacy and pharmacology.

[16] Assimo Poulou AN, Zlatanos SN, Papageorgiou VP (2005). Antioxidant activity of natural resins and bioactive triterpenes in oil substrate. Food chemistry.

[17] Kulkarni RR, Patki PS, Jog VP, Gandage SG, Patwardhan B (1991). Treatment of osteoarthritis with a herbomineral formulation:a double-blind, placebocontrolled, crossover study. Journal of ethnopharmacology.

[18] Krohn K, Rao MS, Raman NV, Khalilullah M (2001). Highperformance thin layer chromatographic analysis of anti-inflammatory triterpenoids from *Boswellia serrata*Roxb. Phytochemical analysis.

[19] Poeckel D, Werz O (2006). Boswellic acids:biological actions and molecular targets. Current medicinal chemistry.

[20] Bakkali F, Averbeck A, Averbeck D, Idaomar M (2008). Biological effects of essential oils-a review. Food and chemical toxicology.

[21] Vlog J, Studela J (1392). Medicinal Plants.

[22] James TK, Rahman A, Douglas JA (1991). Control of Weeds in Five Herb Crops.

[23] Ge F, Zeng F, Liu S, Guo N, Ye H, Song Y, Fan J, Wu X, Wang X, Deng X, Jin Q, Yu L (2010). *In vitro*synergistic interactions of oleanolic acid in combination with isoniazid, rifampicin or ethambutol against *Mycobacterium tuberculosis*. Journal of medical microbiology.

[24] Balcha E, Mengiste B, Gebrelibanos M, Worku A, Ameni G (2014). Evaluation of *in-vitro* anti-mycobacterial activity of selected medicinal plants in Mekelle, Ethiopia. World applied sciences journal.

[25] Wallace Er, Nash DR, Steele LC, Steingrube V (1986). Susceptibility testing of slowly growing mycobacteria by a microdilution MIC method with 7H9 broth. Journal of clinical microbiology.

[26] BD. Middlebrook 7H9 broth with glycerol. Medical technologh, advancing the world of health, December 2006 http://www.bd.com/europe/regulatory/Assets/IFU/US/L007467%2809%29%281206%29.pdf.

[27] Banfi E, Scialino G, Monti-Bragadin C (2003). Development of a microdilution method to evaluate Mycobacterium tuberculosis drug susceptibility. Journal of antimicrobial chemotherapy.

[28] Pagliotto AD, Caleffi-Ferracioli KR, Lopes MA, Baldin VP, Leite CQ, Pavan FR, Scodro RB, Siqueira VL, Cardoso RF (2016). Anti-Mycobacterium tuberculosis activity of antituberculosis drugs and amoxicillin/clavulanate combination. Journal of microbiology, immunology and infection.

[29] Coban AY, Birinci A, Ekinci B, Durupinar B (2004). Drug susceptibility testing of Mycobacterium tuberculosis by the broth microdilution method with 7H9 broth. Memórias do Instituto Oswaldo Cruz.

[30] Labuschagné A, Hussein AA, Rodríguez B, Lall N (2012). Synergistic Antimycobacterial actions of Knowltonia vesicatoria (L.f) sims. Evidence-based complementary and alternative medicine.

[31] Fa G, Fanli Z, Na G, Junwen F, Yu S, Siguo L, Xiuping W, Xuelin W, Xuming D, Qi J, Lu Y (2010). *In vitro*synergistic activity between 8-methoxypsoralen and ethambutol, isoniazid, and rifampin when used in combination against Mycobacterium tuberculosis. World journal of microbiology and biotechnology.

[32] Bapelaa NB, Lall N, Fourie PB, Franzblau SG, Van Rensburg CE (2006). Activity of 7-methyljuglone in combination with antituberculous drugs aginst *Mycobacterium tuberculosis*. Phytomedicine.

